# Comparison of the kinematics, repeatability, and reproducibility of five different multi-segment foot models

**DOI:** 10.1186/s13047-021-00508-1

**Published:** 2022-01-06

**Authors:** Hyo Jeong Yoo, Hye Sun Park, Dong-Oh Lee, Seong Hyun Kim, Gil Young Park, Tae-Joon Cho, Dong Yeon Lee

**Affiliations:** 1 Department of Orthopedic Surgery, Seoul National University Hospital, 101 Daehak-no, Jongno-gu, Seoul, South Korea; 2 Department of Orthopedic Surgery, SNU Seoul Hospital, Seoul, South Korea; 3 Department of Orthopedic Surgery, Seoul National University College of Medicine, 101 Daehak-no, Jongno-gu, Seoul, South Korea

**Keywords:** Multi-segment foot model, Foot kinematics, Repeatability, Reproducibility

## Abstract

**Background:**

Multi-segment foot models (MFMs) for assessing three-dimensional segmental foot motions are calculated via various analytical methods. Although validation studies have already been conducted, we cannot compare their results because the experimental environments in previous studies were different from each other. This study aims to compare the kinematics, repeatability, and reproducibility of five MFMs in the same experimental conditions.

**Methods:**

Eleven healthy males with a mean age of 26.5 years participated in this study. We created a merged 29-marker set including five MFMs: Oxford (OFM), modified Rizzoli (mRFM), DuPont (DFM), Milwaukee (MiFM), and modified Shriners Hospital for Children Greenville (mSHCG). Two operators applied the merged model to participants twice, and then we analysed two relative angles of three segments: shank-hindfoot (HF) and hindfoot-forefoot (FF). Coefficients of multiple correlation (CMC) and mean standard errors were used to assess repeatability and reproducibility, and statistical parametric mapping (SPM) of the t-value was employed to compare kinematics.

**Results:**

HF varus/valgus of the MiFM and mSHCG models, which rotated the segment according to radiographic or goniometric measurements during the reference frame construction, were significantly more repeatable and reproducible, compared to other models. They showed significantly more dorsiflexed HF and plantarflexed FF due to their static offset angles. DFM and mSHCG showed a greater range of motion (ROM), and some models had significantly different FF points of peak angle.

**Conclusions:**

Under the same conditions, rotating the segment according to the appropriate offset angle obtained from radiographic or goniometric measurement increased reliability, but all MFMs had clinically acceptable reliability compared to previous studies. Moreover, in some models, especially HF varus/valgus, there were differences in ROM and points of peak angle even with no statistical difference in SPM curves. Therefore, based on the results of this study, clinicians and researchers involved in the evaluation of foot and ankle dysfunction need an understanding of the specific features of each MFM to make accurate decisions.

**Supplementary Information:**

The online version contains supplementary material available at 10.1186/s13047-021-00508-1.

## Background

There are various multi-segment foot models (MFMs) for assessing three-dimensional foot motion in clinical gait analysis [1]. Each MFM differs not only in the location of the markers on the foot and how the foot segments are defined, but also in the way they are calibrated for the foot’s reference position and coordinate system [2].

Majority of MFMs construct the reference frame using three or more markers placed on each segment identified in the static standing trial. After the static calibration, relative angles of the segments while walking are calculated [3–5]. This is a general marker-based method of performing motion analysis that builds segments by skin-mounted markers, but it is significantly affected by marker-placement errors among sessions or evaluators [5–10]. Some models subtract the static offset values from walking trials [11]. This can increase repeatability and reproducibility, but the omission of anatomical information is a concern [12]. Meanwhile, a few models rotate some coordinate systems according to radiographic and/or goniometric measurements during the reference frame construction [13, 14]. This could be less affected by marker-placement errors and reflect actual bone anatomy. However, this requires subjects to be exposed to radiation, and some measurements are difficult to acquire from radiographic images such as the shank and hindfoot in the transverse plane, and the forefoot in the coronal plane [13].

Previous studies using various MFMs for specific foot deformities can be reviewed and compared by researchers and clinicians [1]; however, these comparisons are limited because it is difficult to understand the special feature of each MFM and its relative differences from other MFMs. Although repeatability studies have already been conducted individually for each model, multiple factors such as the demographic characteristics of the subjects, laboratory environment, operators, statistical analysis, and test intervals were still different, which could have affected their outcomes [5, 7–10, 14–16].

Di Marco, Rossi [12] conducted a comparative study on four MFMs. They identified the most repeatable and reproducible model only in the sagittal plane and the kinematic differences between treadmill and over-ground walking without comparing MFMs. Nicholson, Church [17] also verified, via a comparative study of five MFMs using an amalgamated model, that the MFMs had moderate to low variability as assessed by standard deviations, and that using the same normative data for each model is important when comparing findings between laboratories. However, they did not consider the shank coordinate system that affects the foot kinematics and did not include some methods of applying offset angles, such as radiographic measurements. Consequently, these previous studies have limitations in comparing MFMs simultaneously.

Therefore, this study aimed to compare the kinematics, inter-session repeatability, and inter-evaluator reproducibility among five MFMs of healthy males during walking with all their markers simultaneously in the same experimental conditions with various analytical methods.

## Methods

### Study design

This is a cross-sectional study to analyse the differences in repeatability and reproducibility of five MFMs applied to normal healthy males.

### Participants

Eleven healthy male volunteers with a mean age of 26.5 years participated in this study. The inclusion criteria were as follows: 1) no soft tissue injuries of both feet and ankles within one year from the experimental date, 2) no history of fracture or surgery on both lower extremities, 3) no abnormal findings on both feet radiographs including arthritic changes, 4) no pain during gait. The institutional review board of Seoul National University Hospital approved this study, and all participants provided informed consents prior to participation.

### Multi-segment foot models

**Fig. 1:**
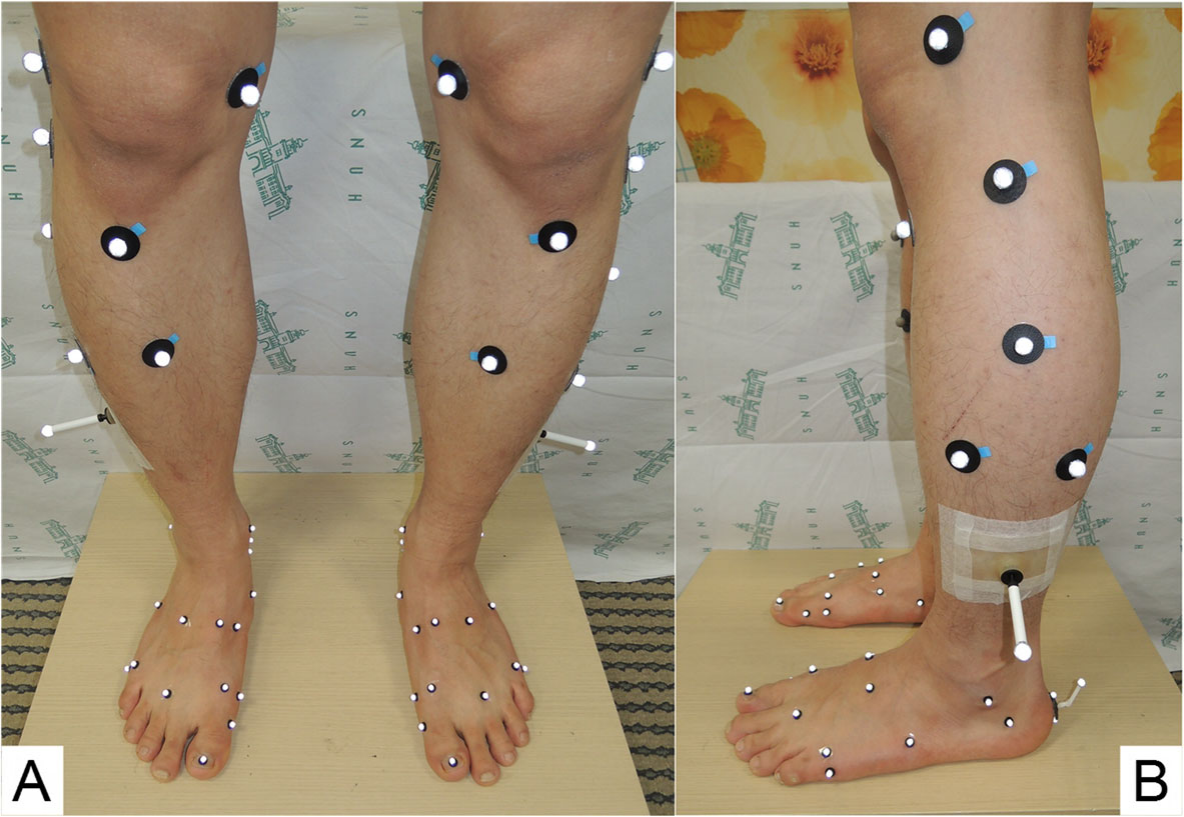
Anterior (**A**) and lateral (**B**) view of a merged 28-marker set

We selected five MFMs which have been validated and used in clinical articles [1]: DuPont foot model (DFM) [3], modified Rizzoli foot model (mRFM) [4], Oxford foot model (OFM) [5], Milwaukee foot model (MiFM) [13], and modified Shriners Hospital for Children Greenville foot model (mSHCG) [14]. We created a merged 28-marker set using nine 8-mm spheres on the shank and twenty 4-mm spheres on the foot (Fig. 1 and Additional file 1). To apply the merged set, we slightly modified the marker placements of mRFM. It attaches the markers on the second metatarsal head and base, but the other models attach these markers between the second and third metatarsal heads and bases. Since it was difficult to place them separately due to their positional difference of only a few millimeters, we placed the markers between the second and third metatarsal heads and bases only. Although hallux is an important segment to be considered during gait, it was excluded because it was too small to allow for the successful attachment of the markers of all the models.

### Data acquisition and post-processing

Two physical therapists with 8 years of experience participated in attaching the merged set to all subjects. A preliminary analysis was done prior to this study to have an appropriate level of experience with other foot models in two subjects. They individually applied the merged 28-marker set to the participants and repeated it after one or two weeks to analyse inter-trial and inter-session repeatability and inter-evaluator reproducibility. To prevent offset angles from influencing foot positioning during static calibration, we maintained the participants’ feet in a fixed position during the first and second visit. Specifically, we marked two parallel straight lines on the ground using masking tape at 30 cm apart, and the center of the subject’s heel and the second toe were placed on the line. After the static calibration, the subjects walked at a self-selected speed along an 8-m walkway. Kinematic data were captured using 12 cameras with a 3D optical motion capture system (Motion Analysis Co., Santa Rosa. CA) and Cortex 1.3.0 software (Motion Analysis Co., Santa Rosa. CA) at a sampling rate of 120 Hz. Visual3D Professional v6.01.36 (C-Motion, Inc.) was used for post-processing and building the five models. According to the original paper of each MFM, the Euler/Cardan angle sequence of OFM, MiFM, and mSHCG was sagittal-coronal-transverse, while DFM and mRFM used the sagittal-transverse-coronal sequence. All kinematic data were time-normalized to 100% of the gait cycle (1% interval between time points), and three representative walking trials were chosen from each session. To use unified terminology, we defined “tibia and shank” as “shank,” “calcaneus and hindfoot” as “hindfoot,” and “metatarsus and forefoot” as “forefoot.” Then, we analysed two relative angles between three segments: shank-hindfoot (HF) and hindfoot-forefoot (FF). We indicated the tracking markers constituting the segments of each model in Additional file 1. To assist understanding of the tri-planar motions, superscripts were used: i.e., HF motion in the sagittal, coronal, and transverse planes were abbreviated as HF^sag^, HF^cor^, and HF^trans^, respectively.

### Radiographic and goniometric measurements

**Fig. 2:**
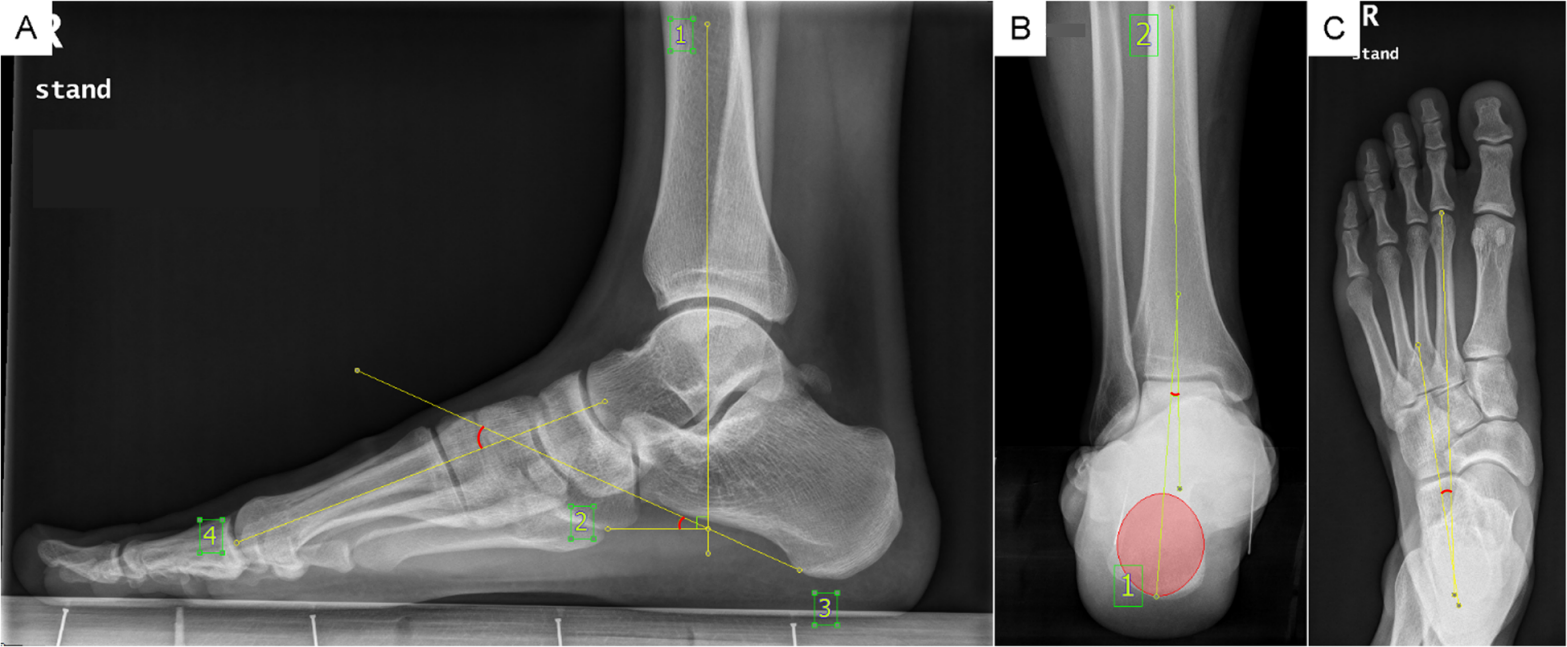
Radiographic measurements from the lateral view (**A**), posterior view (**B**), and standing A/P (**C**)

DFM, mRFM, and OFM used the general marker-based method without subtracting the static offset from the corresponding value of the walking trials, while MiFM used a method of rotating each segment in the medial/lateral, anterior/posterior, and longitudinal axes from radiographic measurements. Thus, the participants underwent three weight-bearing X-ray scans of the dorsoplantar foot, lateral view of the foot and ankle, and heel alignment view (Fig. 2). Two evaluators (physical therapists) individually collected the radiographic measurements according to the original papers’ methods and applied it to each segment rotation [18, 19]. Furthermore, mSHCG rotated the HF segment in the anterior/posterior axis according to goniometric measurements of the HF varus/valgus, and provided options of rotating the FF and HF segments to the pitch angles measured from the radiographic images, or constructing the segments with the markers only. Accordingly, we selected Option 2 for the HF, a radiograph for calcaneal pitch, and Option 3 for the FF, a marker-based FF pitch [14]. The goniometric measurement of HF valgus/varus was taken between the calcaneal axis and the line perpendicular to the floor during weight bearing standing. To reduce the effect of foot posture, we placed the participants’ feet in a fixed position similar to static calibration. Moreover, to minimize the influence of the evaluator’s angle of view during measurement, they were asked to measure the angle with their eyes at the subject’s ankle height.

### Statistical analysis

Coefficients of multiple correlations (CMC) [20] and experimental errors (σ) [21] were calculated to assess inter-trial and inter-session repeatability and inter-evaluator reproducibility. Inter-trial CMC was calculated from two strides processed by one evaluator in the second session and analysed by the averaged data from three strides for each session. To determine inter-evaluator CMC, the averaged data from the second session were selected. Moreover, the inter-trial errors (σ^trial^), inter-session errors (σ^sess^) of each evaluator, and inter-evaluator errors (σ^eval^) were calculated for each model. These were expressed as the mean standard deviation of 0 to 100 points of the whole gait cycle. The Kruskal-Wallis test and Bonferroni correction were performed to find differences in the range of motion (ROM) (α = 0.05). When we analysed CMC and ROM, SPSS statistics 25.0 for windows (SPSS Inc., Chicago, IL) was used. To compare the kinematics of five models, statistical parametric mapping (SPM) [22] of the t-value from the post-hoc unpaired t-test (α = 0.01) was employed using MATLAB (R2019a, The MathWorks, Inc., Natick, MA). SPM of the t-value was used for identifying the difference between continuous curves, which can be calculated using open-source SPM1d code (www.spm1D.org).

## Results

### Repeatability and reproducibility

The inter-trial CMCs of HF and FF were greater than 0.886 in all models (see Table 1). In the sagittal plane, the inter-session and inter-evaluator CMCs of all segments were greater than 0.840 in all models. In HF^cor^ repeatability, the CMCs of DFM, OFM, and mRFM were less than 0.660, whereas those of MiFM and mSHCG were greater than 0.793. For OFM, the averaged inter-session and inter-evaluator CMCs were lower in HF^cor^ (0.424 and 0.410, respectively) than in HF^trans^ (0.810 and 0.791, respectively). Similarly, this was also observed in FF^cor^ (0.467 and 0.612, respectively), when compared to FF^trans^ (0.931 and 0.846, respectively). The repeatability and reproducibility of FF were generally greater than HF in all planes, but in FF^trans^, the reproducibility of mRFM and mSHCG (0.332 and 0.441, respectively) were lower than their repeatability (0.735 and 0.735, respectively).

**Table 1:** Repeatability and reproducibility of five multi-segment foot models

Model	Plane	Shank - Hindfoot								Hindfoot - Forefoot							
		Inter-trial		Inter-session				Inter- evaluator		Inter-trial		Inter-session				Inter- evaluator	
				evaluator A		evaluator B						evaluator A		evaluator B			
		CMC	σ^trial^	CMC	σ^sess^	CMC	σ^sess^	CMC	σ^eval^	CMC	σ^trial^	CMC	σ^sess^	CMC	σ^sess^	CMC	σ^eval^
DFM	Sagittal	0.959	1.2	0.903	2.0	0.950	1.6	0.934	2.0	0.967	0.9	0.869	1.8	0.958	1.2	0.920	1.7
	Coronal	0.926	1.2	0.554	2.5	0.584	2.0	0.652	3.1	0.929	1.1	0.696	2.1	0.606	1.8	0.733	2.9
	Transverse	0.895	1.3	0.461	3.0	0.777	2.1	0.627	3.5	0.886	1.2	0.618	2.3	0.850	1.7	0.837	2.3
mRFM	Sagittal	0.957	1.2	0.957	1.8	0.952	1.7	0.944	2.2	0.983	0.7	0.971	1.3	0.947	1.2	0.929	1.7
	Coronal	0.923	1.0	0.194	2.8	0.660	1.8	0.401	3.4	0.931	0.6	0.862	1.3	0.835	1.3	0.862	1.8
	Transverse	0.943	0.9	0.807	2.1	0.838	1.7	0.778	2.3	0.968	0.6	0.746	1.8	0.723	1.9	0.332	2.7
OFM	Sagittal	0.966	1.0	0.946	1.6	0.960	1.3	0.962	1.6	0.982	0.5	0.934	1.0	0.941	1.0	0.924	1.2
	Coronal	0.923	0.8	0.327	2.2	0.520	2.0	0.410	3.1	0.961	0.6	0.481	2.6	0.453	2.0	0.612	3.4
	Transverse	0.953	0.8	0.752	2.1	0.867	1.5	0.791	2.0	0.988	0.5	0.931	1.2	0.930	1.1	0.846	1.8
mSHCG	Sagittal	0.962	1.1	0.960	1.6	0.962	1.4	0.971	1.7	0.981	0.8	0.926	1.9	0.840	2.4	0.871	2.7
	Coronal	0.929	1.0	0.836	1.7	0.897	1.4	0.742	2.7	0.924	0.8	0.873	1.5	0.663	1.6	0.720	2.0
	Transverse	0.946	0.6	0.734	1.9	0.881	1.2	0.827	1.9	0.974	0.6	0.672	1.9	0.797	1.7	0.441	3.1
MiFM	Sagittal	0.968	1.1	0.933	1.9	0.946	1.6	0.952	2.0	0.987	0.7	0.968	1.2	0.978	1.0	0.965	1.4
	Coronal	0.940	0.8	0.859	1.3	0.793	1.4	0.898	1.8	0.902	0.8	0.702	1.4	0.771	1.2	0.848	1.6
	Transverse	0.971	0.4	0.939	0.8	0.654	1.7	0.728	2.9	0.936	0.6	0.873	1.0	0.931	0.9	0.628	1.8

Coefficients of multiple correlations (CMC) and mean standard errors (σ) of inter-trial (σ^trial^), inter-session (σ^sess^), and inter-evaluator (σ^eval^) of relative motions of the shank-hindfoot and hindfoot-forefoot in five multi-segmental foot models

With respect to the averaged σ, σ^trial^ of all models were less than 1.3° for both HF and FF (Table 1). HF^cor^, DFM, OFM, and mRFM showed σ^sess^ ranging from 1.8° to 2.8° and σ^eval^ ranging from 3.1° to 3.4°, whereas MiFM and mSHCG showed σ^sess^ ranging from 1.3° to 1.7° and σ^eval^ of 1.8° and 1.7°, respectively. FF σ^sess^ ranged from 0.9° to 2.6° among all models, which was generally lower than that of HF, and σ^eval^ ranged from 1.2° to 3.4°, which was higher than σ^sess^. All averaged σ of all models did not exceed 3.5°.

### Kinematics

**Fig. 3:**
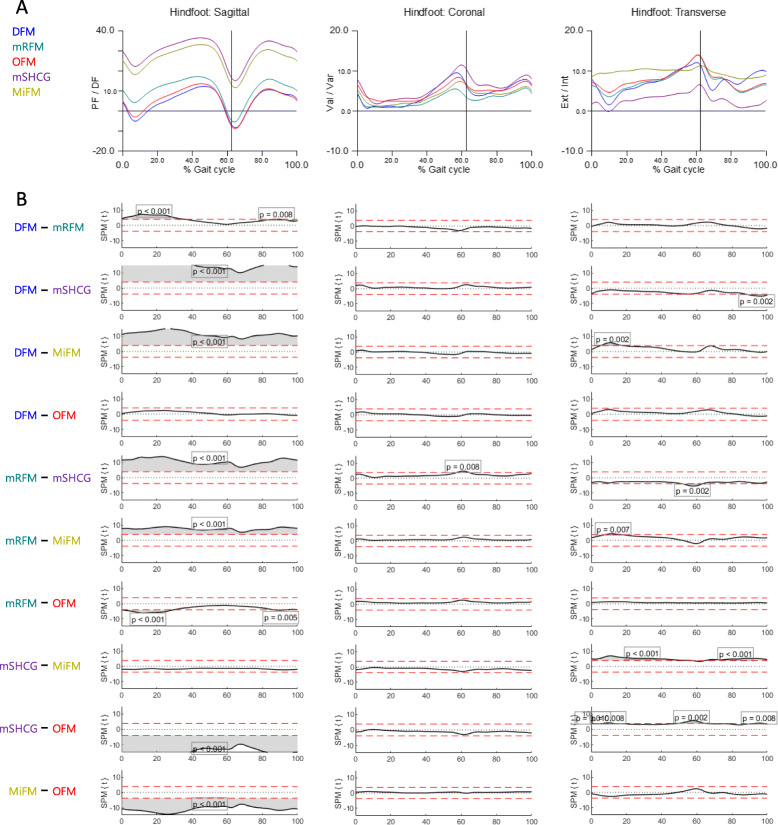
The hindfoot kinematics of five multi-segment foot models. **A** Average graphs for each model (% gait cycle). **B** Differences among the models were visualized by statistical parametric mapping of the t-values from the post-hoc unpaired t-test (α = 0.01)

**Fig. 4:**
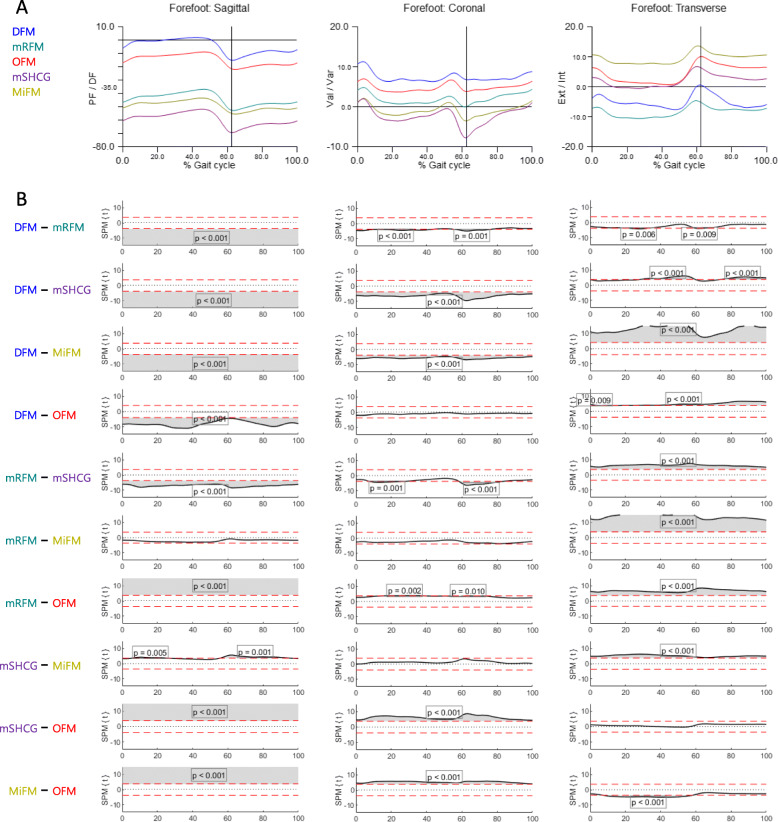
The forefoot kinematics of five multi-segment foot models. **A** Average graphs for each model (% gait cycle). **B** Differences among the models were visualized by statistical parametric mapping of the t-values from the post-hoc unpaired t-test (α = 0.01)

**Fig. 5:**
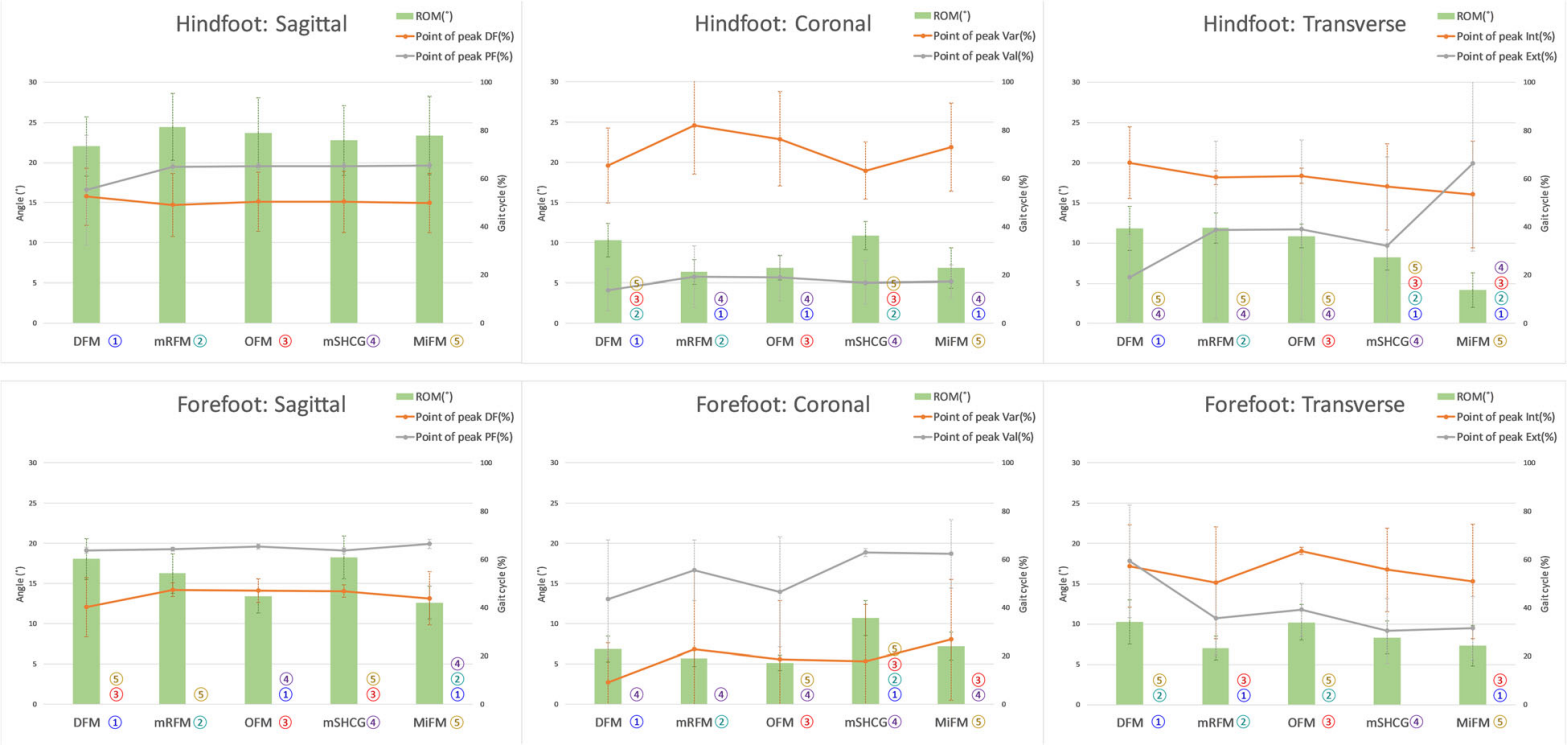
Mean and standard deviations of ROM and the point of peak angle of each MFM. Each MFM was numbered with a different color, and the number of MFM with significant differences in ROM was indicated next to the graph of each MFM. There were significant differences in ROM between MFMs in all motions except “Hindfoot: Sagittal,” and the the peak angle time showed large deviations in the coronal and transverse planes

Figures 3 and 4 show the mean kinematics for each model and SPM curves acquired from the post-hoc unpaired t-test of MFMs. In HF^sag^, MiFM and mSHCG were more dorsiflexed than DFM, OFM, and mRFM in the whole cycle due to static offset angles, and there were no differences in ROM among MFMs (Figs. 3 and 5). In HF^cor^, all SPM curves showed no significant differences except for mRFM-mSHCG, but the DFM and mSHCG had significantly greater ROMs. In HF^trans^, MiFM and mSHCG showed significantly reduced ROM than the others. The SPM curves of HF for DFM-OFM were not statistically different in all planes. In contrast to HF^sag^, FF^sag^ of MiFM, mSHCG, and mRFM were more plantarflexed than those of DFM and OFM in the whole cycle (Fig. 4). In addition, there were significant differences in overall SPM curves for FF kinematics between the models in all planes. With respect to the point of peak angle, each model was significantly different in FF^sag^ and FF^trans^, and some MFMs had standard deviations greater than the mean in HF^trans^ and FF^cor^ (Fig. 5).

## Discussion

In this study, we investigated the kinematic differences, repeatability, and reproducibility of five MFMs that are widely used in the clinical gait analysis and obtained several meaningful results.

According to classifications suggested by Garofalo, Cutti [20], the inter-session repeatability and inter-evaluator reproducibility of all five MFMs in our study ranged from “very good” to “excellent” in the sagittal plane [7–9, 15]. In general gait analysis, the reliability decreased from the sagittal to the coronal, and transverse planes [23, 24], but the reliability of the coronal plane was lower than that of the transverse plane for HF in the OFM and mRFM models [7, 9]. Furthermore, the OFM, DFM, and mRFM models, which have a marker-based analysis, showed a lower repeatability of HF in the coronal plane than in the transverse plane compared to MiFM and mSHCG, which use offset angles. There may be more variables such as the influence of the Euler/Cardan angle sequence [25]; however, we consider that the horizontal variation in the placement of the two posterior heel markers had a major impact on the marker-based analysis of coronal HF motion. Thus, clinicians or researchers using only marker-based analysis should consider using a calcaneal marker placement device [26] or establish a more precise criterion for locating these markers and sticking them carefully.

McGinley, Baker [27] stated in their review that in common clinical situations, an error of 2° or less was highly likely to be acceptable. Errors between 2° and 5° were also likely to be regarded as reasonable but may require consideration in data interpretation. Schwartz, Trost [21] introduced experimental errors in the lower extremities (excluding the multi-segmented foot), and the mean σ^eval^ ranged from 1.2 to 5.3°. In a study that applied σ to MFM, Deschamps, Staes [8] reported that mean σ^sess^ ranged between 0.9° and 5.0°, while the mean σ^eval^ ranged between 2.8° and 7.6°. Saraswat, MacWilliams [14] verified that mean σ^sess^ and σ^eval^ were less than 6.0°. In our study, MiFM and mSHCG showed lower σ than other models in motion analysis with offset angles, but the σ^trial^, σ^sess^, and σ^eval^ of all MFMs did not exceed 3.5°. This indicated that the highest σ in our study was lower than those of previous studies. Therefore, we believe that rotating the segments by the offset angles obtained from radiographic and/or goniometric measurements increased reliability, and consequently avoided the effects of marker-placement errors. However, any model used in our study would be clinically acceptable.

MiFM and mSHCG showed greater HF dorsiflexion and FF plantarflexion compared to other models because they reflected the pitch angles of the calcaneus and FF [13, 14]. Additionally, we verified a close affinity in HF for all planes between OFM and DFM, which corresponds with the findings of Nicholson, Church [17]. However, the CMCs for DFM of HF^trans^ were lower than those for OFM. This suggests that the DFM, by using markers on the medial/lateral malleolus to coordinate the HF segment, was more variable in the transverse plane than the OFM, which applies markers on the medial/lateral calcaneus.

Although most MFMs showed no statistical differences in HF varus/valgus SPM curves except for the toe-off of mRFM-mSHCG, DFM and mSHCG showed significantly increased ROM. In the HF external/internal rotation, there were also similarities in kinematics and ROM in DFM, mRFM, and OFM; however, MiFM and mSHCG showed inconsistent kinematics and decreased ROM compared to the other models. In addition, the point of peak angle showed large deviations in some motions and significant differences in FF. We think that these dissimilarities were not due to the offset angle but to the different local coordinate system and marker placement for each MFM. In particular, soft tissue artifacts that occur differently in each model due to the marker placement discrepancy even within the same segment also had a critical influence [28, 29]. In previous studies, the wand marker, which was used for mSHCG, reflected only 40–70% of the actual axial hip rotation when attached to the lateral thigh [30]. Similarly, the three markers on the lateral shank, which were used for DFM, can rotate themselves by moving with the calf muscle, creating excessive rotation in the proximal segments [17]. In other words, the sensitivity to motion was different for each MFM due to the influence of differences in the segment coordination and the marker type and location. Hence, these factors must be considered when comparing clinical studies using different MFMs.

We obtained meaningful results by comparing the kinematic characteristics of the five models, but it was impossible to find a model that accurately depicts actual foot motions. To compare MFMs with actual foot movements, the influence of STA must be considered. Although there was a study that measured rear, mid, and forefoot kinematics and ROM through an invasive in vivo study using bone pins [31], it could not be compared with our results because the subjects and experimental environments were different. In addition, valuable studies have been conducted to quantify the STA between the skin-mounted marker and the bone to identify the location most affected by STA [32–34]. They reported that the medial malleolus [32], lateral malleolus [34], navicular [32, 33], medial calcaneus [32], lateral calcaneus [33], and posterior aspect of the proximal calcaneus [34] were significantly affected by STA during maximum plantarflexion. In particular, Schallig, Streekstra [34] reported that, in a study with a computed tomography scan, RFM that utilized the posterior aspect of the proximal calcaneus marker as a tracking marker was significantly affected by STA, compared to OFM, which used the proximal calcaneal marker only in the anatomical coordinate system. In summary, although we could not compare the bone movement in five models, further studies are needed to find a model that most accurately reflects actual foot movement.

Some limitations should be considered when appreciating these results. First, only young adult men were analysed in this study. The biomechanics of the elderly, children, and females may differ from those of young males. Second, although the number of subjects in this study was small, it is similar to that of other studies [7, 8, 15, 17]. Further research is required to investigate the implications of the findings to a wider population. Finally, we applied some slight modifications of marker placements to mRFM, OFM, and mSHCG for convenience. This could affect the FF kinematics of mRFM and those of HF of OFM and mSHCG.

## Conclusion

Rotating the segment according to the appropriate offset angle obtained from radiographic or goniometric measurements increased reliability. However, even with kinematic similarities, ROMs and the point of peak angle were different for each MFM. Therefore, it was impossible to define an MFM close to the actual foot and ankle motions in this study, but it is important to consider that different MFMs have different reliability and sensitivity to motion when understanding clinical findings. Clinicians and researchers involved in the evaluation of foot and ankle dysfunction need an understanding of the specific features of each MFM to make accurate decisions. Based on the results of this study, further studies are needed to determine which model closely reflects the actual foot and ankle motions.

## Supplementary Information

**Additional file 1.** Table. Names and anatomical landmarks of a 28-merged marker set.

## Data Availability

The datasets used and/or analysed during the current study are available from the corresponding author on reasonable request.

## Abbreviations

MFMs: Multi-segment foot models
DFM: DuPont foot model
mRFM: Modified Rizzoli foot model
OFM: Oxford foot model
MiFM: Milwaukee foot model
mSHCG: Modified Shriners Hospital for Children Greenville foot model
HF: The relative motion of shank-hindfoot
FF: The relative motion of hindfoot-forefoot
CMC: Coefficients of multiple correlations
σ: Experimental errors
σ^trial^: Inter-trial errors
σ^sess^: Inter-session errors
σ^eval^: Inter-evaluator errors
SPM: Statistical parametric mapping
