# Supplementary material for: Comparison of the kinematics, repeatability, and reproducibility of five different multi-segment foot models
Source: J Foot Ankle Res. 2022 Jan 6;15:1. doi: 10.1186/s13047-021-00508-1 (PMC8734222; doi:10.1186/s13047-021-00508-1)
Supplement: Supplementary file 1 — Additional file 1. Table. Names and anatomical landmarks of a 28-merged marker set. [file 13047_2021_508_MOESM1_ESM.docx]

Table. Names and anatomical landmarks of a 28-merged marker set.

| No. | Maker name | Description | DFM | OFM | MiFM | mSHCG | mRFM |
| --- | --- | --- | --- | --- | --- | --- | --- |
| 1 | KL | Lateral femoral condyle | S* | S* |  | S* |  |
| 2 | KM | Medial femoral condyle | S* |  |  | S* |  |
| 3 | TIBU | Tibial tuberosity, relatively upper |  | S |  | S | S |
| 4 | TIBL | Anterior tibia, medial side |  | S | S | S |  |
| 5 | HOF | Head of fibular |  | S |  |  | S |
| 6 | TOP | Lateral shank (triad) | S |  |  |  |  |
| 7 | FRONT | Lateral shank (triad) | S |  |  |  |  |
| 8 | REAR | Lateral shank (triad) | S |  |  |  |  |
| 9 | TIBW | Distal lateral tibia (wand) |  |  |  | S |  |
| 10 | ANKL | Lateral malleolus | S*, H | S | S | S | S |
| 11 | ANKM | Medial malleolus | S*, H* | S* | S | S* | S* |
| 12 | CALP | Center of proximal aspect of heel | H | H* | H | H | H |
| 13 | CALD | Center of distal aspect of heel | H | H |  |  | H |
| 14 | CALW | Wand maker placed midpoint  between CALD and CALP |  | H |  |  |  |
| 15 | CALA | Lateral aspect of calcaneus at the same distance from the most posterior point as TALI |  | H | H | H | H |
| 16 | TALI | Sustentaculum tali |  | H | H | H | H |
| 17 | NAVI | Most prominence aspect of navicular bone | F |  |  |  |  |
| 18 | M1B | The base of 1^st^ metatarsal |  | F |  | F | F |
| 19 | M1BM | Medial aspect of the base of 1^st^ metatarsal |  |  |  | F* |  |
| 20 | M23B | Between bases of 2^nd^ and 3^rd^ metatarsals |  |  |  | F* | F** |
| 21 | M5B | The base of 5^st^ metatarsal | F | F | F |  | F |
| 22 | M1H | Head of 1^st^ metatarsal | F |  |  | F | F |
| 23 | M1HM | Medial aspect of the head of 1^st^ metatarsal |  | F* | F | F* |  |
| 24 | M23H | Between heads of 2^nd^ and 3^rd^ metatarsals | F | F |  | F* | F** |
| 25 | M5H | Head of 5^st^ metatarsal | F |  |  | F | F |
| 26 | M5HL | Lateral aspect of the head of 5^st^ metatarsal |  | F | F |  |  |
| 27 | HALB | Proximal phalanx of the hallux |  | Hx |  |  | Hx |
| 28 | HALN | midpoint of hallux nail bed | Hx |  |  |  |  |

S: one of the markers that consist of the shank.
H: one of the markers that consist of the hindfoot.
F: one of the markers that consist of the forefoot.
Hx: one of the markers that consist of the hallux (not used for analysis).
*: a static trial only.
**: a different placement from the original placement.
